# Persistent Lymphopenia as a Poor Prognostic Factor in Patients With Multiple Organ Dysfunction Syndrome in the Renal Intensive Care Unit: A Retrospective Single‐Center Study

**DOI:** 10.1002/iid3.70152

**Published:** 2025-02-14

**Authors:** Xiang Xue, Shuhua Zhu, Shutian Xu, Yuchao Zhou, Yang Wang, Lixuan Lou, Shijun Li

**Affiliations:** ^1^ National Clinical Research Center of Kidney Disease Nanjing Medical University, Jingling Hospital Nanjing China

**Keywords:** immunosuppression, intensive care units, lymphopenia, multiple organ dysfunction syndrome, survival

## Abstract

**Purpose:**

Multiple organ dysfunction syndrome (MODS), defined as two or more organ dysfunction during infection or following shock or trauma, correlates with poor outcomes. Clinical data, including MODS in the renal intensive care unit (ICU), are scarce. Therefore, we investigate the clinical characteristics and prognosis of patients with MODS in the renal ICU.

**Methods:**

A single‐center, retrospective cohort study of 99 adult patients with MODS admitted to the renal ICU of the National Clinical Research Center of Kidney Disease, Jinling Hospital, Nanjing, China, from October 1, 2011 to October 1, 2021.

**Results:**

99 patients had a mean age of 49.7 ± 16.5 years old, and 51 (51.5%) patients died within 28 days after being admitted to the renal ICU. Infection (80 patients, 80.8%) was the most common reason for admission, with 47 cases being pulmonary infections. Of all of the 99 patients, 73 (73.7%) presented with persistent lymphocytopenia (lymphocyte count < 1.1 × 10^9^/L from the day of ICU admission through to day 7), with 33 and 40 presenting moderate (lymphocyte count 0.6–1.1 × 10^9^/L) and severe persistent lymphopenia (lymphocyte count ≤ 0.6 × 10^9^/L), respectively. These patients had higher illness severity and chronic kidney disease (CKD) prevalence. Patients with severe persistent lymphopenia were associated with higher 28‐day ICU mortality (87.5% vs. 42.4% vs. 7.7%, *p* < 0.001) versus those with moderate and without persistent lymphopenia. Multivariable logistic regression analysis revealed that the number of organs involved, APACHE‐II score, and persistent lymphopenia were independent risk factors for 28‐day mortality in patients with MODS. The value of lymphocyte count on day 7 of admission in predicting poor prognosis of patients was higher than on other days (Area Under Curve, AUC = 0.831).

**Conclusions:**

Patients with MODS are critically ill with high mortality. Persistent lymphopenia is frequent in patients with MODS and is independently associated with 28‐day mortality. Lymphocyte counts on day 7 of admission were shown to be highly predictive of prognosis.

## Graphical abstract

1. Patients with multiple organ dysfunction syndrome(MODS) are critically ill with high mortality in the renal intensive care unit (ICU). 2. Patients with persistent lymphocytopenia are more likely to be treated with mechanical ventilation, high dose vasoactive drugs, to be pathogen‐positive and at high risk of death, especially those with severe persistent lymphocytopenia. 3. Lymphocyte counts on day 7 of admission were shown to be highly predictive of prognosis.

## 1 Introduction

Multiple organ dysfunction syndrome (MODS) constitutes 10% of admissions to the intensive care unit (ICU), with mortality rates varying from 30% to 70%, imposing an enormous burden of high healthcare costs. The prognosis of patients with MODS predominantly depends on the number of organs involved, the extent of organ damage and the duration of the illness [1, 2, 3]. MODS is commonly accompanied by uncontrolled immune system dysfunction, caused by the production of damage‐associated molecular patterns (DAMPs) following substantial tissue damage and ischemia [4, 5]. Despite recent advances in critical care management strategies, patients’ prognosis have not improved significantly. Enhanced comprehension of the immune systems underlying severe illness in MODS patients might lead to better outcomes.

As an essential part of the immune system, the reduced number and function of lymphocytes further weaken the body's ability to resist infection. Several diseases can cause lymphopenia, increasing the risk of adverse prognosis [6, 7]. A recent study has linked early lymphopenia following trauma to an increased risk of death, with the 48‐h lymphocyte count being the strongest predictor [8]. According to another study, persistent lymphopenia on day 4 following bacteremia diagnosis shows both early and late mortality in patients with sepsis [9]. Children with severe sepsis and persistent lymphopenia are at risk of prolonged MODS or Pediatric ICU mortality [10]. Previous studies on MODS have mainly focused on emergency or surgical ICUs, whereas there are limited studies on MODS patients in the renal ICU. Therefore, in this study, we retrospectively analyzed the clinical characteristics and prognostic factors associated with MODS patients admitted to the renal ICU and evaluated the 28‐day mortality based on the lymphocyte count at admission and its progression on day 7.

## 2 Materials and Methods

### 2.1 Study Population

This retrospective database cohort study was conducted at the National Clinical Research Center of Kidney Disease, Jinling Hospital, Nanjing, China. Patient database information is collected through two primary methods: automated computer systems and manual data entry performed by clinicians. This study was reviewed and approved by the Ethical Committee of Nanjing Jinling Hospital (no. 2022DZKY‐033‐01). Due to the retrospective nature of the study, informed consent from the patients was waived.

### 2.2 Study Protocol

**Figure 1:**
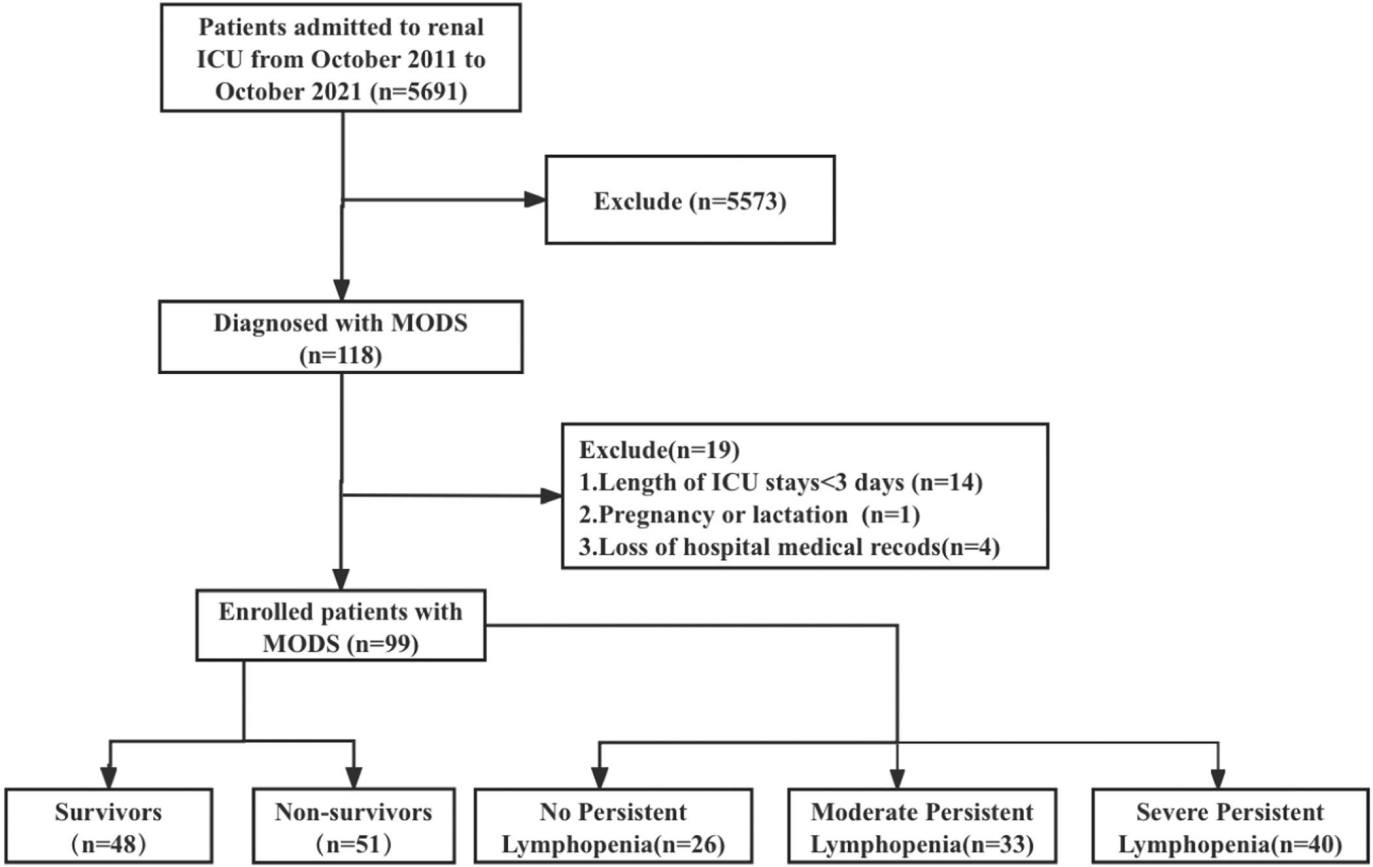
Screening process for MODS patients in renal ICU. MODS, multiple organ dysfunction syndrome; ICU, intensive care unit.

Among 5691 patients admitted to the renal ICU of our center from October 1, 2011 to October 1, 2021, 99 MODS patients were involved. All patients met the diagnostic criteria of MODS, defined as the acute development of dysfunction in two or more organs according to the MODS score by Marshall et al. (Supplementary Table 1) [11, 12]. Exclusion criteria included: (1) Length of ICU stays < 3 days; (2) Pregnancy or lactation; (3) Loss of hospital medical records. The enrolled patients were divided into two groups: the survival group and the non‐survival group. Furthermore, patients were divided into three cohorts according to lymphocyte counts within 7 days of admission to the renal ICU: (1) patients with lymphocyte counts within the normal range (≥ 1.1 × 10^9^/L); (2) patients with moderate persistent lymphopenia (0.6–1.1 × 10^9^/L); (3) patients with severe persistent lymphopenia (≤ 0.6 × 10^9^/L) (Figure 1).

The primary analysis was a multivariable analysis of the clinical factors independently associated with 28‐day mortality. Secondary outcomes included the duration of hospital and renal ICU stays, the use of vasopressors, mechanical ventilation, and continuous renal replacement therapy during hospitalization.

### 2.3 Study Variables

The database gathered baseline characteristics, including patients' demographics such as age, sex, baseline comorbidities, number of organs involved, acute physiology and chronic health evaluation‐II (APACHE‐II) score, the sequential organ failure assessment (SOFA) score, source of infection, and measures of organ support (mechanical ventilation, vasopressors, and renal replacement therapy). Laboratory data included lymphocyte count, neutrophil‐lymphocyte ratio, systemic immune‐inflammatory index (SII) [13], hemoglobin values, albumin, bilirubin, serum creatinine (SCr), ALT, NT‐pro BNP, and Interleukin‐6. Infection is defined as the invasion of pathogens (bacteria, viruses, fungi, parasites, etc.) into the body, resulting in localized tissue and systemic inflammation in the host. The diagnosis and classification of AKI and CKD were based on the Kidney Disease Improving Global Outcomes criteria [14, 15]. Baseline creatinine levels were established as the average creatinine measurements obtained within 3 months before admission. When pre‐morbid reference SCr is unavailable, the estimated baseline SCr could be back‐calculated from the glomerular filtration rate (GFR) = 75 mL/min/1.73 m^2^ using the Modification of Diet in Renal Disease (MDRD) study equation, the so‐called “eGFR 75 approach” [16]. The criteria used for the definition of CKD in adults are: (1) signs of kidney damage, most often determined by an elevated urine albumin‐to‐creatinine ratio (ACR); or (2) reduced kidney function, indicated by GFR < 60 ml/min per 1.73 m^2^ (Supplementary Table 2).

### 2.4 Statistical Analyses

Data were analyzed using IBM SPSS Statistics 26.0 (SPSS Inc., Chicago, IL, USA) and GraphPad Prism (GraphPad Software, San Diego, California, USA). Qualitative data are shown as numbers (percentage), while measurement data are expressed as mean ± standard deviation or median ± interquartile range. A one‐way analysis of variance was utilized to compare quantitative continuous variables between groups. Categorical variables were compared with the chi‐square test. Cumulative survival curves were constructed with the Kaplan‐Meier method, and statistical differences between survival curves were evaluated using the log‐rank test. Logistic regression analysis was used for univariable analysis, and variables with *p* < 0.1 were put into multivariate models for screening using stepwise regression analysis. The reliability of using main clinical variables to predict death due to MODS was calculated by plotting receiver operating characteristic (ROC) curves, and the optimal diagnostic cut‐off value was determined according to the point with the largest Youden index. Confidence intervals on the area under ROC curves (AUCs) were calculated using non‐parametric assumptions. The level of significance was set at *p* < 0.05.

## 3 Results

### 3.1 Patients Information

A total of 99 MODS patients were included in this study, 51 patients (51.5%) died within 28 days after being admitted to the renal ICU. 73 patients (73.7%) presented with persistent lymphocytopenia, with 33 presenting moderate and 40 presenting severe persistent lymphopenia, respectively. Infection (80.8%) was the most frequent reason for hospitalization, followed by wounds from snakes or wasps (6.1%) and heatstroke (6.1%). The non‐survivor group had a higher severity of illness with more organ failures, increasing APACHE–II score, SOFA score, NT‐proBNP, and Interleukin‐6 (*p* < 0.05). The lymphocyte, albumin, and CD4^+^T lymphocyte levels in the non‐survivor group were lower than in the survivor group (*p* < 0.05). However, neither the age nor the sex of the two groups varied significantly (Tables 1 and 2).

**Table 1:** Characteristics and outcomes of patients in the survivor group and non‐survivor group.

Variables	Survivors (*n* = 48)	Non‐survivors (*n* = 51)	*P*
Age(years), mean (SD)	46.9 ± 14.0	52.4 ± 18.4	0.097
Sex(male), *n* (%)	33 (68.8)	33 (64.7)	0.670
APACHE‐II score, median (IQR)	16.0 (12.3, 19.0)	29.0 (26.0, 32.0)	< 0.001
SOFA score, median (IQR)	9.0 (6.3, 11.0)	15.0 (11.4, 18.0)	< 0.001
Number of organs involved, median (IQR)	3 (3, 4)	4 (4, 5)	< 0.001
Etiology of admission			
Infection	35 (72.9)	45 (88.2)	0.053
Snake bites, bee stings	5 (10.4)	1 (2.0)	0.105
Heatstroke	4 (8.3)	2 (3.9)	0.427
Others	4 (8.3)	3 (5.88)	0.710
Acute on chronic kidney diease, *n* (%)	7 (14.6)	40 (78.4)	< 0.001
Acute kidney injury, *n* (%)	41 (85.4)	11 (25.6)	< 0.001
Comorbidities, *n* (%)			
Coronary artery disease	2 (4.2)	3 (5.9)	1.0
Congestive heart failure	4 (8.3)	11 (21.6)	0.066
Cerebrovascular disease	4 (8.3)	2 (3.9)	0.427
Hypertension	14 (29.2)	18 (35.3)	0.515
Diabetes	9 (18.8)	13 (25.5)	0.420
Liver disease	2 (4.2)	8 (15.7)	0.093
Chronic kidney disease	7 (14.6)	40 (78.4)	< 0.001
Post‐renal transplatation	1 (2.1)	14 (27.5)	< 0.001
Lupus nephritis	3 (6.3)	7 (13.7)	0.320
Membranous nephropathy	0	4 (7.8)	0.118
Minimal change disease	0	2 (3.9)	0.495
Diatebic nephropathy	1 (2.1)	2 (3.9)	1.0
IgA nephropathy	1 (2.1)	0	0.485
Others	0	5 (9.8)	0.057
No renal biopsy	1 (2.1)	6 (11.8)	0.113
Site of infection, *n* (%)			
Respiratory	17 (35.4)	30 (58.8)	0.020
Others	10 (20.8)	15 (29.4)	0.326
Type of infection, *n* (%)			
Gram‐positive	6 (12.5)	2 (3.9)	0.152
Gram‐negative	10 (20.8)	22 (43.1)	0.018
Mixed	3 (6.3)	5 (9.8)	0.716
Fungal	2 (4.2)	19 (37.3)	0.001
Culture‐negative	1 (2.1)	7 (13.7)	0.060
Laboratory variables at ICU admission, median (IQR)			
WBC count(×10^9^/L)	12.4 (7.2, 21.6)	15.0 (9.0, 24.4)	0.332
Lymphocyte count(×10^9^/L)	0.72 (0.43, 1.14)	0.49 (0.25, 0.68)	0.002
Platelet count(×10^9^/L)	60.0 (29.0, 147.0)	52.0 (35.0, 95.0)	0.305
NLR	11.3 (5.1, 31.2)	26.9 (15.0, 47.5)	0.007
SII	677.4 (208.5,1997.9)	2132.4 (1195.0, 3426.7)	< 0.001
Albumin (g/L)	29.0 ± 5.8	26.1 ± 5.5	0.012
Total bilirubin(μmol/L)	31.8 (18.1, 67.45)	27.6 (11.9, 57.6)	0.294
ALT(U/L)	131.0 (69.0, 403.0)	75.0 (34.0, 544.0)	0.063
Serum creatinine (μmol/L)	234.8 (144.2, 440.0)	194.0 (116.0, 344.5)	0.183
NT‐pro BNP (pg/mL)	872.5 (199.4, 2001.5)	1979.0 (450.0, 4138.0)	0.033
Interleukin‐6(ng/L)	79.4 (24.3, 230.4)	227.5 (82.0, 441.0)	0.004
CD4^+^T lymphocyte count (pcs/μL)	296.0 (130.0, 494.0)	104.0 (44.0, 236.0)	< 0.001
Clinical Outcome			
ICU LOS, median number of days (IQR)	9.5 (7.0, 15.0)	12.0 (6.0, 17.0)	0.726
Hospital LOS, median number of days (IQR)	19.0 (13.0, 22.8)	14.0 (7.0, 17.0)	0.001
Vasopressor use, *n* (%)			
Low dose	11 (22.9)	28 (54.9)	0.001
High dose or multiple	2 (4.2)	20 (39.2)	< 0.001
Mechanical ventilation, *n* (%)	6 (12.5)	40 (78.4)	< 0.001
Duration of ventilation, median number of days (IQR)	3.5 (1.0, 4.2)	7.0 (5.2, 12.0)	0.002
Renal replacement therapy, *n* (%)	40 (83.3)	44 (86.3)	0.068

*Note:* SD, standard deviation; IQR, interquartile range; APACHE‐II score, acute physiology and chronic health evaluation‐II score; SOFA score, sequential organ failure assessment score; NLR, neutrophil‐lymphocyte ratio; SII, systemic immune‐inflammatory index; LOS, length of stay; Low dose of norepinephrine use, < 0.5 μg/kg/min; High dose of norepinephrine use, ≥ 0.5 μg/kg/min.

### 3.2 Conditions of Chronic Kidney Disease

Out of the total number of patients, 47 had a previous diagnosis of CKD (11 CKD Stage G1; 10 CKD Stage G2; 13 CKD Stage G3; 6 CKD Stage G4; 2 CKD Stage G5 receiving peritoneal dialysis and 5 receiving hemodialysis). Among these cases, there were 15 instances after kidney transplantation, 10 cases of lupus nephritis, 4 cases of membranous nephropathy, 2 cases of minimal change disease, 3 cases of diabetic nephropathy, 1 case of IgA nephropathy, and 5 cases of others (2 renal amyloidosis, 1 multiple myeloma‐related renal impairment, 1 cryoglobulinemic glomerulonephritis, 1 polycystic kidney disease), in addition to 7 CKD patients without renal biopsies. Forty‐one individuals were administered immunosuppressive treatment 3 months before their hospitalization. Oral prednisone was taken on average 25.6 ± 16.1 mg/d, with a median duration of 14 months (interquartile range, 4.5–57 months). Additional immunosuppressive therapy included pulse methylprednisolone (27.3%), mycophenolate mofetil (20.1%), tacrolimus (16.2%), tripterygium glycosides (9.1%), cyclophosphamide (6.1%), and cyclosporine (4.0%). The duration of immunosuppressive treatment was 13 months (interquartile range, 5–58.5 months). Five individuals received oral prednisone only (Table 1).

**Table 2:** Characteristics and outcomes of patients categorized by lymphocyte count.

Variables	No Persistent Lymphopenia (*n* = 26)	Moderate Persistent Lymphopenia (*n* = 33)	Severe Persistent Lymphopenia (*n* = 40)	*P*
Age(years), mean (SD)	48.2 ± 14.4	52.9 ± 15.7	48.1 ± 18.4	0.531
Sex(male), *n* (%)	20 (76.9)	19 (57.6)	27 (67.5)	0.231
APACHE II score, median (IQR)	14.0 (12.0, 17.0)a*	23.0 (16.0, 29.0)	28.0 (25.5,32.0)c*	< 0.001
SOFA score, median (IQR)	8.0 (5.0, 11.0)a*	12.0 (10.0, 15.5)	15.0 (14.0,17.0)c*	< 0.001
Number of organs involved, median(IQR)	3 (3,4)	4 (3,4)b*	4 (4,5)c*	< 0.001
Comorbidities, *n* (%)				
Coronary artery disease	1 (3.8)	3 (9.1)	1 (2.5)	0.519
Congestive heart failure	0a*	9 (27.3)	6 (15)	0.015
Cerebrovascular disease	0	3 (9.1)	3 (7.5)	0.324
Hypertension	6 (23.1)	14 (42.4)	12 (30)	0.265
Diabetes	4 (15.4)	11 (33.3)	7 (17.5)	0.167
Chronic kidney disease	4 (15.4)	14 (42.4)b*	29 (72.5)c*	< 0.001
Liver disease	3 (11.5)	1 (3.0)	6 (15)	0.180
Site of infection, *n* (%)				
Bacteremia	2 (7.7)	3 (9.1)	1 (2.5)	0.496
Respiratory	4 (15.4)	15 (45.5)	28 (70.0)c*	< 0.001
Gastrointestinal	3 (11.5)	3 (9.1)	2 (5.0)	0.502
Skin and soft tissue	0	1 (3.0)	2 (5.0)	0.781
Multiple	1 (3.8)	3 (9.1)	4 (10.0)	0.727
Type of infection, *n* (%)				
Gram‐positive	3 (11.5)	3 (9.1)	2 (5.0)	0.651
Gram‐negative	3 (11.5)a*	14 (42.4)	15 (37.5)	0.028
Mixed	1 (3.8)	3 (9.1)	4 (10.0)	0.727
Fungal	1 (3.8)	4 (12.1)b*	16 (40.0)c*	0.001
Culture‐negative	1 (3.8)	2 (6.1)	5(12.5)	0.464
Laboratory variables at ICU admission, median (IQR)				
WBC count (×10^9^/L)	12.7 (6.95‐25.2)	15.6 (10.2‐22.8)	13.5 (6.8,21.7)	0.641
Platelet count (×10^9^/L)	64.0 (28.5, 187.2)	49.0 (30.5, 143.1)	51.0 (36.0, 105.5)	0.351
NLR	7.8 (4.0, 17.9)a*	18.8 (14.0, 43.6)	31.0 (11.7, 55.1)c*	< 0.001
SII	466.3 (152, 2055.4)	1335.4 (548.7, 2237.9)	2263.4 (996.7, 4392.4)c*	0.001
Albumin (g/L)	27.6 ± 7.1	28.9 ± 5.3	26.2 ± 5.0	0.145
Total bilirubin(μmol/L)	31.8 (19.3, 55.6)	43.0 (15.9, 80.0)	19.7 (10.5, 56.3)	0.230
ALT(U/L)	109.5 (70.5, 458.0)	150.5 (50.5, 761.0)	76.5 (33.3, 364.0)	0.153
Serum creatinine (μmol/L)	217.0 (169.0, 367.8)	275.7 (128.0, 470.5)	172.8 (116.0, 321.0)	0.114
NT‐pro BNP (pg/mL)	636.1 (143.1, 3535.5)	1778.0 (468.8, 4134.0)	1042.0 (438.8, 3387.4)	0.186
Interleukin‐6(ng/L)	50.0 (18.0, 164.4)	188.0 (58.4, 386.0)	204.0 (55.0, 412.3)c*	0.023
CD4^+^T lymphocytes (pcs/μL)	348.5 (237.2, 545.3)	276.0 (139.5, 379.0)b*	73.0 (31.5, 127.8)c*	< 0.001
Clinical Outcome				
ICU LOS, median number of days (IQR)	8.0 (6.8, 11.8)	12.0 (7.0, 15.0)	14.0 (6.3, 18.0)	0.105
Hospital LOS, median number of days (IQR)	17.5 (12.8, 20.5)	15.0 (8.0, 22.0)	15.0 (7.0, 18.8)	0.251
Vasopressor use, *n* (%)				
low dose	4 (15.4)a*	15 (45.5)	20 (50)c*	0.013
high‐dose or multiple	3 (11.5)	5 (15.2)	14 (35)c*	0.04
Mechanical ventilation, *n* (%)	2 (7.7)	11 (33.3)b*	33 (82.5)c*	< 0.001
Renal replacement therapy, *n* (%)	22 (84.6)	30 (90.1)	40 (80)	0.436
Death, *n* (%)	2 (7.7)a*	14 (42.4)b*	35 (87.5)c*	< 0.001

*Note:* SD, standard deviation; IQR, interquartile range; APACHE‐II score, acute physiology and chronic health evaluation‐II score; SOFA score, sequential organ failure assessment score; NLR, neutrophil‐lymphocyte ratio; SII, systemic immune‐inflammatory index; LOS, length of stay; Low dose of norepinephrine use, < 0.5 μg/kg/min; High dose of norepinephrine use, ≥ 0.5 μg/kg/min.

a *: There were statistically significant differences in the characteristics or outcomes between patients without persistent lymphopenia and those with moderate persistent lymphopenia.

b *: There were statistically significant differences in the characteristics or outcomes between patients with moderate and severe persistent lymphopenia.

c *: There were statistically significant differences in the characteristics or outcomes between patients with severe and without persistent lymphopenia.

### 3.3 Etiology of Infection

The non‐survival group was more likely to suffer from respiratory infections than the survival group (58.8% vs. 35.4%, *p* = 0.020). No significant difference was observed in the areas of the skin and soft tissue, bloodstream, gastrointestinal, or any other infection sites. In contrast to the survival group, the proportion of gram‐negative bacteria and fungi was significantly higher in the non‐survival group. The major pathogens included Acinetobacter baumannii, *Klebsiella pneumoniae*, Pseudomonas aeruginosa, *Escherichia coli*, Pneumocystis carinii, and Aspergillus (Table 1).

### 3.4 Organs Involvement

**Figure 2:**
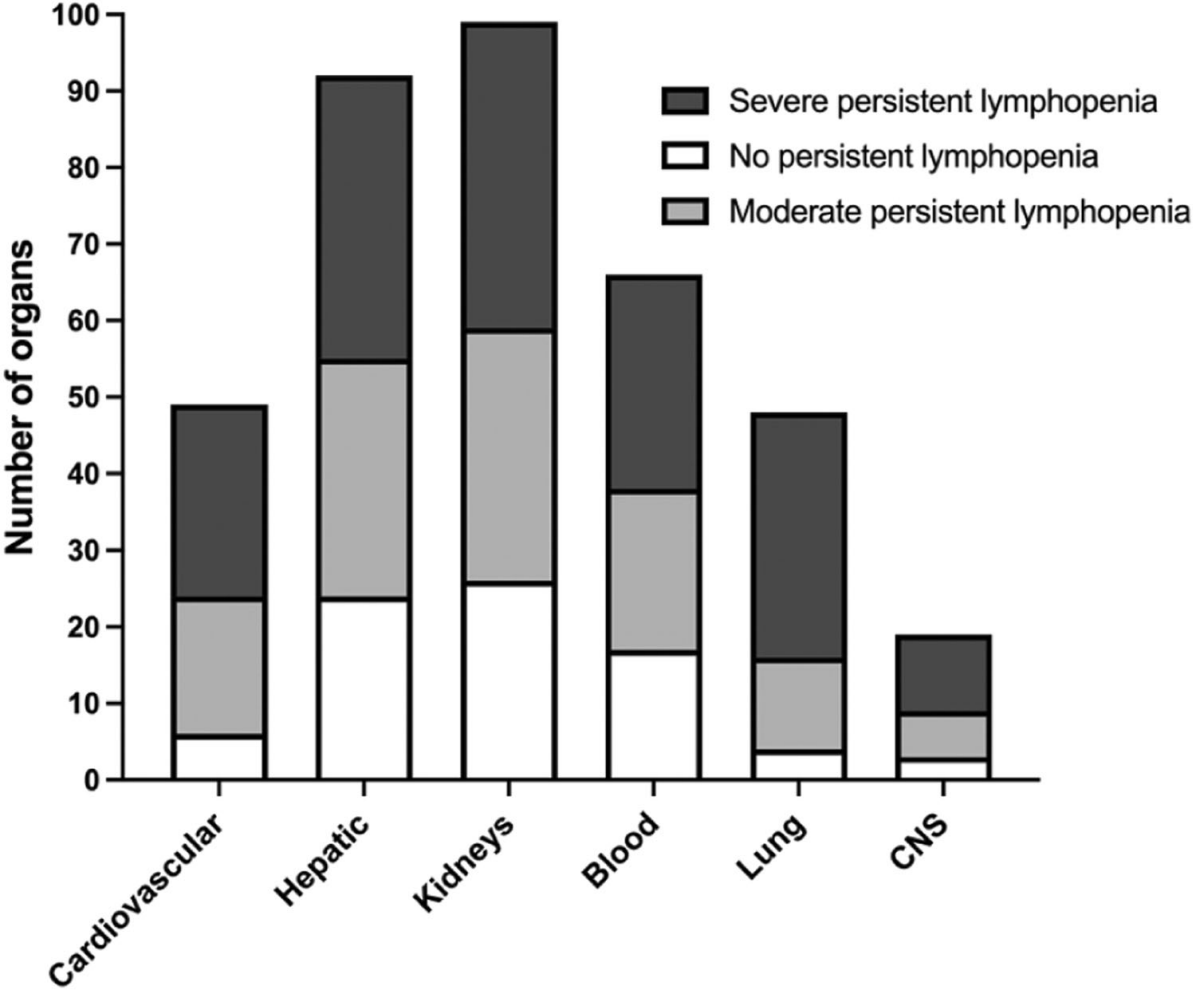
Number of organs/systems involved according to the severity of lymphopenia. CNS, center nervous system.

There was a strong correlation between the fatalities of patients with MODS and the number of organs affected. In particular, patients with dysfunction in four or more organs had a mortality rate above 60%. The most frequently involved organs and systems were the kidneys (100%), followed by the hepatic (92.9%), hematological system (66.7%), cardiovascular system (49.5%), lung (48.5%) and central nervous system (19.2%). The proportion of cardiovascular system (23.1% vs. 54.5% vs. 62.5%, *p* = 0.006) and lung (15.4% vs. 36.4% vs. 80%, *p* < 0.001) involvement was notably higher in patients with severe persistent lymphopenia (Figure 2).

### 3.5 Clinical Outcomes

Eighty‐four patients (84.8%) received CRRT after admission. Blood pressure was maintained by fluid rehydration, colloid infusion, and vasoactive drugs in patients with hemodynamic instability. Thirty‐nine patients (39.4%) received a low dose of norepinephrine (< 0.5 μg/kg/min) to maintain blood pressure, and 22 patients (22.2%, 2 patients in the survivor group vs. 20 patients in the non‐survivor group) required a high dose of norepinephrine (≥ 0.5 μg/kg/min) or a combination of vasoactive agents. All patients required oxygen therapy after admission, of which 46 patients (46.5%, 6 patients in the survivor group vs. 40 patients in the non‐survivor group) required mechanical ventilation. There were statistically significant differences in the number of patients treated with mechanical ventilation and vasoactive drugs between the two groups, while there was no statistically significant difference in the number of patients treated with CRRT (Table 1).

**Figure 3:**
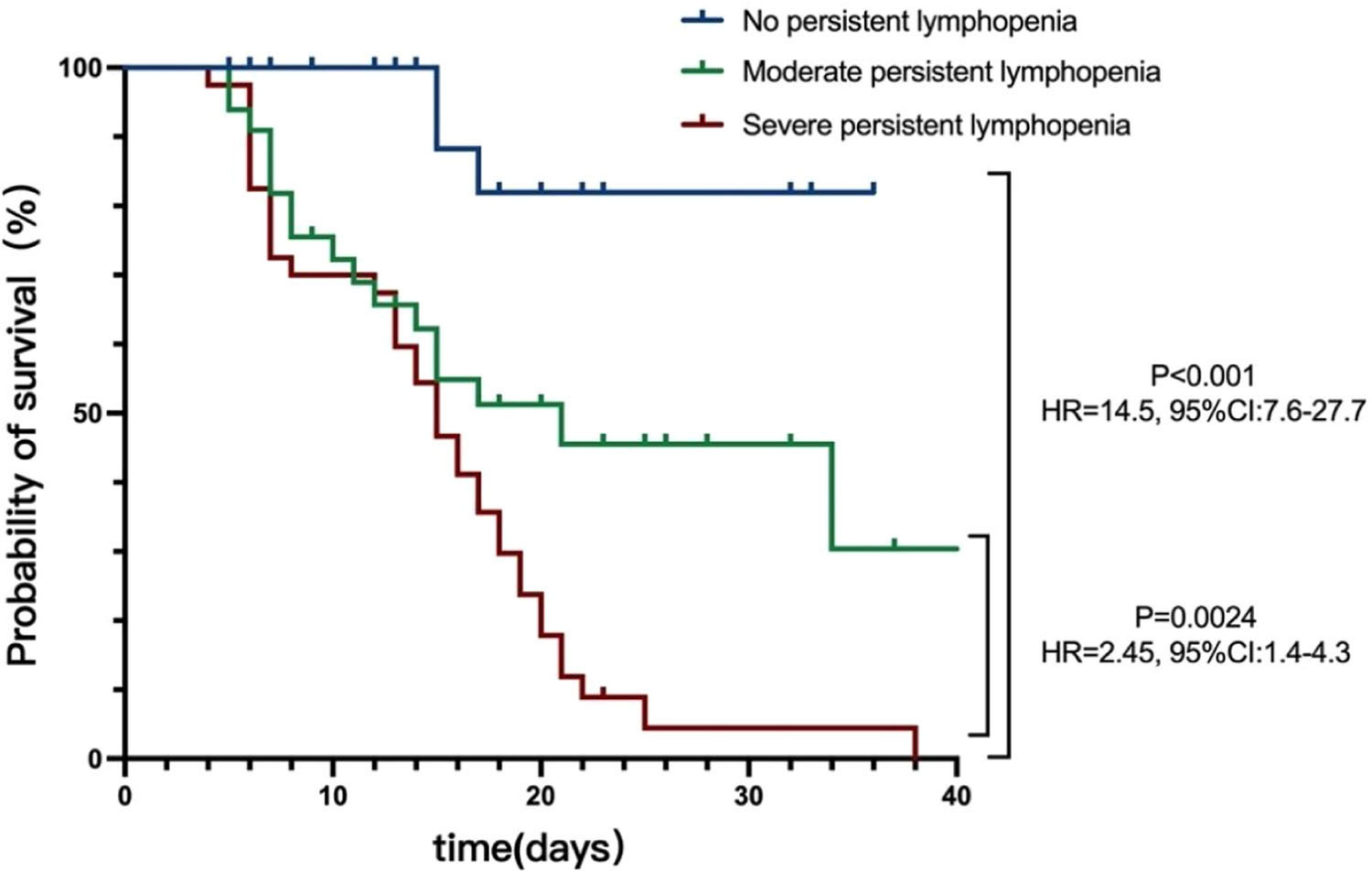
Unadjusted Kaplan‐Meier survival analysis according to absolute lymphocyte counts.

Survival worsened as the degree of lymphocytopenia increased. The 28‐day mortality in patients with severe persistent lymphopenia group was 87.5%, moderate persistent lymphopenia was 42.4% (42.4% vs. 87.5%, *p* = 0.0024), whereas in patients without persistent lymphopenia was 7.7% (7.7% vs. 87.5%, *p* < 0.001). The survival curves of the three groups are shown in Figure 3.

### 3.6 Prognostic Factor Analysis

Univariable logistic regression analysis revealed that age, the number of organs involved, SOFA score, APACHE‐II score, hemoglobin, albumin, NT‐proBNP, CD4^+^T lymphocytes, and persistent lymphocytopenia were risk factors for mortality in MODS patients (*p* < 0.1). Multivariable Logistic regression analysis demonstrated that the number of organs involved, APACHE‐II score, and persistent lymphocytopenia were independent predictors of death in patients with MODS (Table 3).

**Table 3:** A Risk factors for 28‐day death in MODS patients.

Variables	Univariate	*P*	Multivariate	*P*
	HR(95% CI)		HR(95% CI)	
Age	1.088 (0.996, 1.047)	0.099		
Sex	1.200 (0.519, 2.774)	0.670		
Number of organs involved	5.980 (2.868, 12.464)	< 0.001	5.159 (1.340, 19.853)	0.017
SOFA score	2.021 (1.544, 2.645)	< 0.001		
APACHE‐II score	1.537 (1.308, 1.806)	< 0.001	1.594 (1.244, 2.043)	< 0.001
NLR	1.015 (0.089, 1.032)	0.089		
Albumin (g/L)	0.911 (0.844, 0.983)	0.016		
NT‐proBNP (pg/mL)	1.000 (1.000, 1.001)	0.019		
Interleukin‐6 (ng/L)	1.000 (1.000, 1.001)	0.313		
CD4^+^T lymphocytes (pcs/μL)	0.994 (0.991, 0.997)	< 0.001		
Persistent lymphopenia	18.812 (6.269, 56.451)	< 0.001	17.794 (1.940, 163.222)	0.011

*Note:* SOFA score, sequential organ failure assessment score; APACHE‐II score, acute physiology and chronic health evaluation‐II score; NLR, neutrophil‐lymphocyte ratio; Persistent lymphopenia, lymphocyte count < 1.1 × 10^9^/L from the day of ICU admission through to day 7.

**Figure 4:**
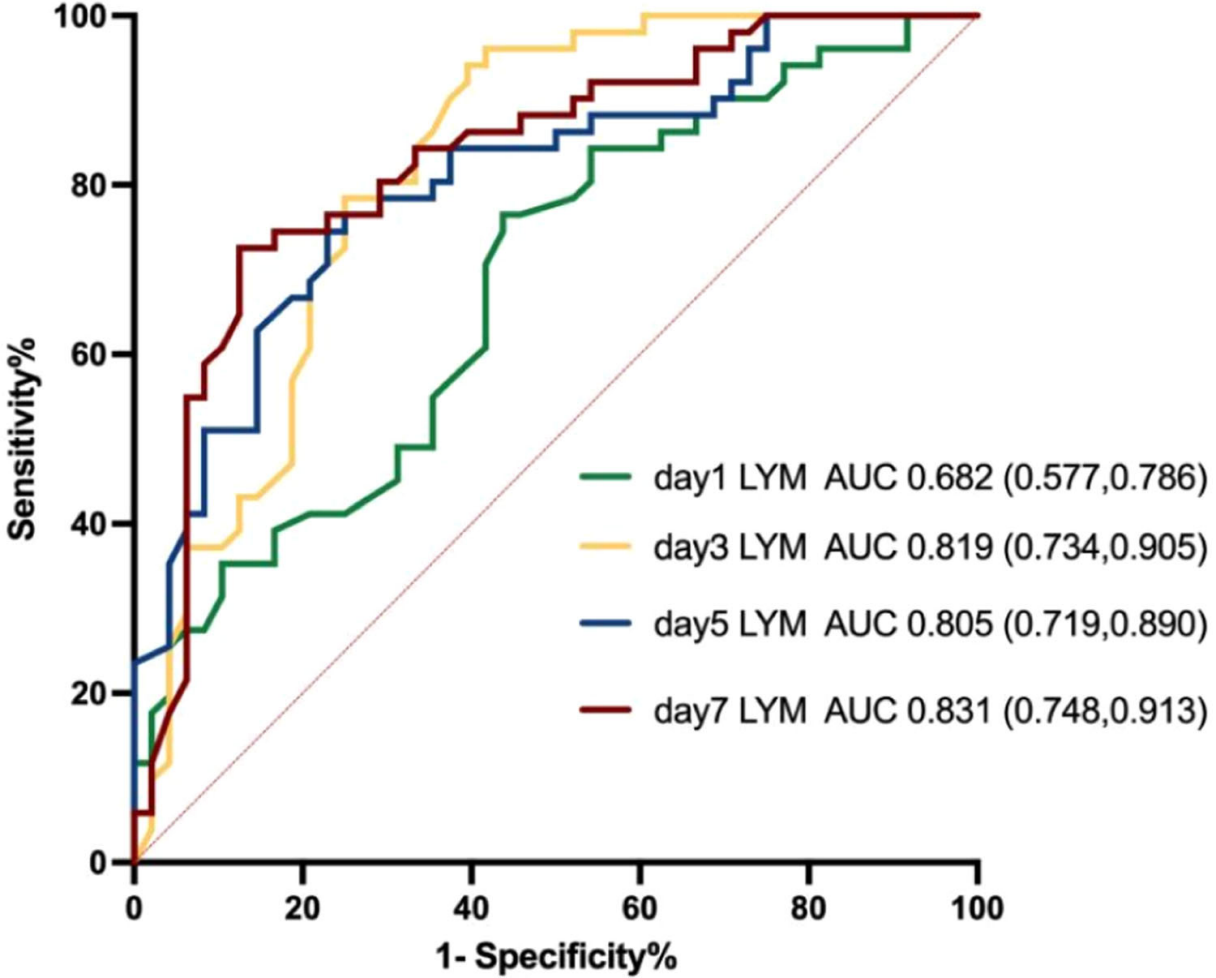
Receiver operator characteristic (ROC) plot evaluating the capacity of lymphocyte counts to predict 28‐day mortality.

The predictive value of various indicators for the 28‐day prognosis of patients with MODS was investigated, and the results showed that the lymphocyte count had a high clinical predictive value. The AUC of the lymphocyte count on day 7 was more significant than on day 1, day 3, and day 5. The day 7 lymphocyte count accurately predicts patient prognosis with an appropriate cut‐off value of 0.58 × 10^9^/L (87.5% sensitivity, 72.5% specificity) (Figure 4).

## 4 Discussion

MODS is a common complication of systemic inflammatory response syndrome and represents the terminal stage of sepsis development. It can affect multiple systemic organs and progress rapidly, often leading to life‐threatening admissions to the intensive care units [17]. The leading cause of MODS in our study was infection, followed by poisoning and heatstroke. The kidneys were the organs most frequently involved in this study. Previous studies have revealed that patients with AKI are more prone to develop sepsis [18, 19, 20]. AKI progressing to CKD is a crucial contributor to organ dysfunction and high mortality [21, 22]. Renal tubule epithelial cells are extremely vulnerable to oxidative stress. DAMPs, produced by necrotic tubule epithelial cells, elicit a local inflammatory response and distant systemic effects on other organs via PPR toll‐like receptors (TLRs) [23, 24, 25].

The total number of lymphocytes routinely detected in clinical practice. Our research found that lymphopenia was commonly observed in patients with MODS during the first 7 days after renal ICU admission. Persistent lymphopenia was associated with higher mortality and was an independent predictor of death. The cut‐off lymphocyte count for predicting 28‐d mortality in patients with MODS was 0.58 × 10^9^/L. After adjusting for confounders, patients with persistent lymphopenia were 17.794 times more likely to die than those without persistent lymphopenia.

Previous studies have revealed that the absolute lymphocyte count and proportion in the total number of leukocytes decline as illness severity and immunosuppression worsen [26, 27]. We studied 40 patients with severe persistent lymphopenia, 33 patients with moderate persistent lymphopenia, and 26 without persistent lymphopenia who were admitted to the renal ICU. The three cohorts observed substantial variations in the baseline characteristics, treatment modalities, and prognosis. The risk of mortality within 28 days following ICU admission was 14.5 times higher in patients with severe persistent lymphocytopenia than in those without persistent lymphocytopenia and 2.45 times higher in those with moderate persistent lymphocytopenia.

Infection was present in 80.8% of patients in this study, with respiratory infection being the most common. The gram‐negative bacterial and fungal infection rate was substantially higher in patients with severe persistent lymphopenia. The top 3 bacterial cultures were Acinetobacter baumannii, *Klebsiella pneumoniae*, *Staphylococcus aureus*, and Pneumocystis carinii accounted for the highest proportion of fungal culture. In patients with granulomatosis with polyangiitis (GPA), pretreatment total lymphocyte count < 0.8 × 10^9^/L and a 3‐month treatment lymphopenia < 0.6 × 10^9^/L were substantially linked to an elevated incidence of Pneumocystis jiroveci pneumonia [28]. In contrast to other critically ill studies, many patients in our study received long‐term immunosuppressant therapy, including high‐dose glucocorticoids or multiple immunosuppressant combinations. These treatments inhibit the activation, proliferation, and survival of macrophages, T lymphocytes, and other inflammatory cells and promote T cell apoptosis. The subsequent macrophage dysfunction and further reduction in T lymphocytes with severe infection cause patients to enter immune paralysis and increase the incidence of opportunistic infection [29, 30].

For secondary outcomes, persistent lymphopenia was deeply linked to an increased likelihood of treatment with mechanical ventilation and vasoactive drugs. Our finding is consistent with the results observed by Derick Adigbli in a multicentre retrospective study conducted in Australia [27]. Since nearly all patients received CRRT in this cohort, this cohort is ill suited for determining risk factors for need for CRRT.

Our study suggests that the real‐time monitoring of lymphocyte counts may help evaluate the prognosis of patients in critical condition. Clinicians should be alerted to the potential risk of severe lymphopenia and asked to take appropriate actions, such as tapering corticosteroids as recommended by current guidelines. The relationship between CD4^+^T lymphopenia and risk of death in our study suggests that measuring CD4^+^T lymphocyte counts in critically ill patients may be useful to gauge the risk of infection and overall prognosis. Still, additional data are needed before recommending routine measurement of CD4 counts in critically ill patients.

Our study also has some limitations: First, due to the small sample size and retrospective nature, the clinical effects of an altered immune response could not be properly corrected for confounding factors. Second, because the primary diseases involved in the study are complex, there may be bias in the factors affecting prognosis. Third, since we did not measure particular lymphocyte subsets, we were unable to determine the extent of lymphopenia present in each lymphocyte subset. Fourth, it's still uncertain if persistent lymphocytopenia is a genuine risk factor for a poor prognosis or just a sign of severe illness. As a single‐center retrospective observational study, these findings should be considered hypothesis‐generating rather than evidence of a causative relationship between lymphopenia and poor outcomes. In the future, expanding the sample size and conducting prospective studies is necessary to overcome the abovementioned limitations.

In conclusion, patients with MODS are not rare in our center and are critically ill with a poor prognosis. This study has demonstrated that patients with persistent lymphocytopenia are more likely to be treated with mechanical ventilation, high dose vasoactive drugs, to be pathogen‐positive and at high risk of death, especially those with severe persistent lymphocytopenia. These results suggest that real‐time monitoring of lymphocyte count may be an early useful marker of immune status and disease severity in patients with MODS and kidney disease in the ICU.

## Author Contributions

**Xiang Xue:** formal analysis, validation, visualization, writing–original draft. **Shuhua Zhu:** funding acquisition, methodology, resources, writing–review and editing. **Shutian Xu:** data curation, validation. **Yuchao Zhou:** writing–review and editing. **Yang Wang:** data curation, visualization. **Lixuan Lou:** investigation, visualization. **Shijun Li:** conceptualization, methodology, resources, supervision, writing–review and editing.

## Supporting information

Supporting information.

Supporting information.

## Data Availability

I confirm that my article contains a Data Availability Statement.
