# Supplementary material for: Persistent Lymphopenia as a Poor Prognostic Factor in Patients With Multiple Organ Dysfunction Syndrome in the Renal Intensive Care Unit: A Retrospective Single‐Center Study
Source: Immun Inflamm Dis. 2025 Feb 14;13(2):e70152. doi: 10.1002/iid3.70152 (PMC11828737; doi:10.1002/iid3.70152)
Supplement: Supplementary file 2 — Supporting information. [file IID3-13-e70152-s002.docx]

**Supplementary Table 1 The Multiple Organ Dysfunction Score**

| Organ system | 0 | 1 | 2 | 3 | 4 |
| --- | --- | --- | --- | --- | --- |
| Respiratory^a^ |  |  |  |  |  |
| (*P*O_2_/*F*iO_2_ ratio) | ≥300 | 226-300 | 151-225 | 76=150 | ≤75 |
| Renal,^b^ serum creatinine |  |  |  |  |  |
| μmol/L | ≤100 | 101-200 | 201-350 | 351-500 | ≥500 |
| mg/dl | ≤1.0 | 1.1-2.0 | 2.1-3.5 | 3.5-5.0 | ≥5.0 |
| Hepatic, serum bilirubin |  |  |  |  |  |
| μmol /L | ≤20 | 21-60 | 61-120 | 121-240 | ≥240 |
| mg/dl | ≤1.0 | 1.1-3.1 | 3.1=6.0 | 6.1-12.0 | ≥12.0 |
| Cardiovascular^c^ |  |  |  |  |  |
| Pressure-adjusted heart rate(PAR) | ≤10.0 | 10.1-15.0 | 15.1-20.0 | 20.1-30.0 | ≥30.0 |
| Hematologic |  |  |  |  |  |
| Platelet count(×10^9^/L) | ≥120 | 81-120 | 51-80 | 21-50 | ≤20 |
| Neurologic^d^ |  |  |  |  |  |
| Glasgow Coma Score | 15 | 13-14 | 10-12 | 7-9 | ≤6 |

a: The *P*O_2_/*F*iO_2_ ratio is calculated without reference to the use or mode of mechanical ventilation and without reference to the use or level of PEEP.

b: The serum creatinine level is measured without reference to the use of dialysis.

c: The pressure-adjusted heart rate (PAR) is calculated as the product of the heart rate and right atrial (central venous) pressure, divided by the mean arterial pressure: PAR = (Heart rate × RAP)/MAP.

d: The Glasgow Coma Score in the patient receiving sedation or muscle relaxants is assumed normal unless there is evidence of intrinsically altered mentation.

**Supplement Table 2 CKD staging according to GFR and urine ACR**

| **CKD Stage** | **Measurement** | **Terms** |
| --- | --- | --- |
| GFR category | GFR (ml/min per 1.73 m^2^) |  |
| G1 | ≥90 | Normal or high |
| G2 | 60–89 | Mildly decreased |
| G3a | 45–59 | Mildly to moderately decreased |
| G3b | 30–44 | Moderately to severely decreased |
| G4 | 15–29 | Severely decreased |
| G5 | <15 | Kidney failure |
| ACR category | Urine ACR (mg/g) |  |
| A1 | <30 | Normal to mildly increased |
| A2 | 30–300 | Moderately increased |
| A3 | >300 | Severely decreased |

CKD, chronic kidney disease; GFR, glomerular filtration rate; ACR, albumin-to-creatinine ratio.
